# Optimizing Artificial Diet Composition for Enhanced Development and Fertility of *Amblyseius swirskii*

**DOI:** 10.3390/insects16111105

**Published:** 2025-10-30

**Authors:** Karlygash Alpysbayeva, Ainura Adilkhankyzy, Assel Seitzhan, Kanat Anuarbekov, Balzhan Naimanova, Shyryn Turbekova

**Affiliations:** 1 Department of Biological Plant Protection, Kazakh Research Institute of Plant Protection and Quarantine Named After Zh. Zhiembaev, Kultobe 1, 050070 Almaty, Kazakhstan; adilkhankyzy.ainura@gmail.com (A.A.); asselseitzhan@mail.ru (A.S.); baljan-sun93@mail.ru (B.N.); shyrynka_turbekova@mail.ru (S.T.); 2 Department of Water Resources and Land Reclamation, Kazakh National Agrarian Research University, Abay 8, 050010 Almaty, Kazakhstan; alshyn-kanat@list.ru

**Keywords:** artificial diet, insect eggs, biological plant protection, phytoseiid mites, mass rearing

## Abstract

The predatory mite *Amblyseius swirskii* is widely used as a biological control agent against phloem-feeding pests in vegetable production. However, its mass rearing is constrained by the lack of standardized and cost-effective artificial diets (ADs). In this study, the effects of various AD compositions on the development and viability of *A. swirskii* were assessed at lab-scale. A basal diet was supplemented with eggs of *Sitotroga cerealella*, *Galleria mellonella*, *Tetranychus turkestani*, and decapsulated *Artemia salina* cysts. The feeder mite *Carpoglyphus lactis* served as a control. Developmental duration, survival, adult longevity, feeding intensity, and fertility were evaluated. The shortest pre-imaginal development (2.9 ± 0.1 days) and highest survival (78%) were observed on *S. cerealella* eggs, while the longest adult lifespan (10.5 ± 0.3 days) and weakest survival (38%) occurred with *A. salina*. In contrast, *G. mellonella* eggs proved to be the least suitable, resulting in minimal survival and reproduction. Study findings support *S. cerealella* and *A. salina* as promising AD components, with further optimization needed for improving reproductive output.

## Simple Summary

The predatory mite *Amblyseius swirskii* is widely utilized in greenhouse crop protection programs to control phloem-feeding pests such as whiteflies, thrips, and aphids. A major challenge in the large-scale rearing of this bioagent lies in the selection of a suitable diet. Conventional rearing relies on natural prey such as the feeder mite *Carpoglyphus lactis*, which is costly and not always feasible for industrial-scale production. In this pioneering study, the performance of *A. swirskii* when maintained on artificial diets supplemented with eggs of various insect and mite species was evaluated. The results demonstrated that the highest developmental and survival rates were achieved with grain moth (*Sitotroga cerealella*) eggs, while the longest adult lifespan was observed with *Artemia salina* cysts. In contrast, wax moth (*Galleria mellonella*) eggs proved unsuitable as a nutritional source. These findings contribute to the optimization of artificial diet formulations, offering a cost-effective and scalable solution for the mass rearing of *A. swirskii*, with long-term benefits for integrated pest management and reduced reliance on chemical pesticides in greenhouse systems.

## 1. Introduction

In modern agriculture, technologies for protected cultivation have advanced considerably, enabling year-round production of vegetables, berries, and ornamentals under controlled conditions [1,2]. While traditional crops such as cucumber, tomato, and pepper remain central, the diversity of greenhouse crops now includes eggplant, leafy greens, strawberry, melon, and numerous ornamental species [2]. Although over 1200 vegetable species are known, fewer than 80 are widely cultivated, and their yields are often reduced by phytophagous pests [3]. Globally, around 100 species of invertebrate pests damage crops in protected systems, decreasing both yield and quality [4].

Greenhouse microclimates, while favorable for plant growth, also accelerate the reproduction of pests such as whiteflies, spider mites, aphids, and thrips [5,6]. In Kazakhstan, Turkestan spider mite (*Tetranychus turkestani*), thrips (Thysanoptera), and whiteflies (Aleyrodidae) are particularly widespread, frequently causing large-scale outbreaks [7,8]. Chemical pesticides are still commonly used, including some banned in the EU and USA but permitted in Kazakhstan [9,10,11]. However, their overuse leads to pesticide residues, resistance development, and reduced efficacy.

As an alternative, biological methods are gaining recognition worldwide [12,13,14,15,16]. Predatory mites such as *Phytoseiulus persimilis* and *Amblyseius swirskii* (Phytoseiidae) are effectively applied against spider mites and thrips [17]. Other species, including *Neoseiulus cucumeris*, *N. barkeri*, *T. montdorensis*, *N. californicus*, and *A. andersoni*, are also widely used in integrated pest management (IPM) of protected crops [18,19,20,21,22,23,24,25,26,27]. In Kazakhstan, however, biological protection remains at an early stage of adoption, hindered by limited farmer awareness, insufficient regulation, and lack of governmental support [28,29,30,31,32,33].

A prerequisite for large-scale application of biocontrol is the efficient mass production of predatory mites. Conventional rearing on host plants infested with phytophages has drawbacks, including high space requirements, unstable reproductive outcomes, and occupational health risks from allergens and microorganisms [14,15]. Thus, the development of artificial diets (ADs) for *A. swirskii* is of particular interest. Yet, challenges remain in formulating nutritionally balanced diets that sustain survival, development, and fertility across generations. Nguyen et al. [34,35] and Riahi et al. [36] demonstrated that diet composition strongly influences mite performance. Protein-rich ingredients (e.g., egg powder, hemolymph) enhance survival, while vitamins and antioxidants improve stress resistance; conversely, imbalanced formulations reduce reproductive output [37,38].

Optimizing artificial feeding systems can reduce costs, ensure stable biocontrol agent production, and strengthen the sustainability of protected cultivation in Kazakhstan. However, the mass rearing of *Amblyseius swirskii* remains challenging, as it requires maintaining nutritional balance, high survival, and reproductive potential across successive generations. In Kazakhstan, this direction is novel and holds significant promise. Building upon international research and successful practices, our objective is to establish a domestic mass-production line for *A. swirskii*, thereby ensuring the availability of effective biological control agents.

## 2. Materials and Methods

### 2.1. Rearing of Amblyseius swirskii and Its Prey Organisms

The laboratory population of *Amblyseius swirskii* was maintained in the entomoacariphage collection of the Kazakh Research Institute of Plant Protection named after Zh. Zhiembaev. This colony has been continuously maintained in the laboratory since 2018 on its traditional diet, namely the dried fruit mite *Carpoglyphus lactis* L. For prey rearing, wheat bran supplemented with sterilized crushed dried fruits was used as the substrate [39]. Rearing conditions were 23–26 °C, 75–85% relative humidity, and a 16:8 hlight:dark photoperiod. The initial density of mites in the bran was 100 individuals per cm^3^, with a substrate layer of 5 cm [27]. Although the colony has not been refreshed with wild individuals, it remains stable under these rearing conditions. At the same time, we are currently investigating alternative artificial diets for *A. swirskii* for its mass production.

For the experiments, we used eggs of *Artemia salina*, *Sitotroga cerealella*, *Galleria mellonella*, and *Tetranychus turkestani*, as these species are routinely maintained in our laboratory as prey sources for other natural enemies and are therefore readily available.

*Artemia salina.* Decapsulated eggs (cysts) of the crustacean *A. salina*, which are freed from their outer chorionic shell, were commercially (Almaty, Kazakhstan) sourced from a local pet supply store.

*Sitotroga cerealella.* To obtain *S. cerealella* eggs under laboratory conditions, mechanized lines were used for the mass production of grain moth eggs. Larvae were reared on barley grain as the primary substrate.

*Galleria mellonella.* The greater wax moth was reared under laboratory conditions in a thermostat at a temperature of 28 °C and relative humidity of 70–80%. Larvae were manually selected and placed in metal sieves with a mesh size of 0.2–0.3 cm. A layer of nutrient medium was placed at the bottom of each container. To optimize pupation, the environment was maintained at a temperature of 28–30 °C, relative humidity of 60–70%, and a photoperiod of 12:12 h (light:dark). After pupation, the larvae formed cocoons, from which adults emerged within 7–10 days. The butterflies laid eggs on filter paper affixed to the outside of the sieve. Eggs were collected using a sterile instrument as they accumulated. For disinfection, the eggs were treated with a 0.5% sodium hypochlorite solution (NaClO) for 2–3 min, followed by three rinses with sterile distilled water (dH_2_O). After treatment, the eggs were dried on sterile paper at room temperature and stored at 4 °C until further use in experiment.

*Tetranychus turkestani.* Bean plants (*Phaseolus vulgaris* L.) were used as a host plant for rearing *T. turkestani*. Bean seeds were pre-germinated in commercial garden soil (Ogorodnik Universal Soil, Almaty, Kazakhstan) in 0.3 L plastic containers. Once the plants reached the stage of 3–5 true leaves, they were infested with an initial population of *T. turkestani* (50 adult individuals per plant). Infested plants were maintained in insectaria under controlled conditions: temperature 25.0 ± 2 °C, relative humidity 65–75%, and a photoperiod of 16:8 (light:dark). To ensure a continuous food supply and population stability, portions of the mite population were transferred to fresh plants every 7–10 days. Regular microscopic examinations were performed to monitor the species composition and prevent contamination or mixing with other *Tetranychidae* species.

Polyfloral pollen. Collected from the flowers of various plant species and rich in vitamins and microelements, was commercially sourced from Honeybee Company (Almaty, Kazakhstan). After purchase, the pollen was finely ground using a laboratory grinder to ensure homogeneity and subsequently sterilized in a drying oven at 80 °C for 30 min prior to incorporation into the artificial diet.

### 2.2. Rearing Conditions of Amblyseius swirskii

In the present study, peptone, yeast extract, dried milk, and phytophage eggs or cysts (*A. salina*, *S. cerealella*, *G. mellonella*, and *T. turkestani*) were used as protein sources in the artificial diets (ADs).

*A. swirskii* individuals were reared on black plastic platforms (5.0 cm × 6.0 cm × 0.3 cm) placed on a 1 cm thick layer of Oasis floral foam, which was submerged in water inside 100 mm × 20 mm glass Petri dishes. The edges of the rearing arenas were lined with water-soaked tissue paper to maintain humidity and prevent mite escape.

A 1.5 cm piece of sewing thread was positioned in the center of each oviposition arena. 10 (ten) phytophage eggs or cysts were added to each arena according to the experimental treatment and coated with AD syrup. Subsequently, one female and one male *A. swirskii* were introduced into each arena. Oviposition threads were replaced every two days. Threads containing phytoseiid eggs were transferred to new platforms for continued observation and assessment of the laid egg viability. The diet was replenished as it was consumed or dried, and each experimental variant was sprayed with the corresponding AD syrup.

### 2.3. Preparation of Artificial Diets

Experiments to evaluate the development and reproduction of the predatory mite *A. swirskii* were conducted using the eggs of various phytophagous species (*S. cerealella*, *G. mellonella*, *C. lactis*, *T. turkestani*) in combination with an artificial diet. Decapsulated cysts (eggs) of *A. salina* were also included as an experimental variant.

The AD base was adapted from Nguyen et al. [35] and Ogawa & Osakabe [37], with modifications to suit our laboratory conditions. It consisted of honey (5%) and sucrose (5%) as carbohydrate sources, tryptone (5%) and yeast extract (10%) as protein- and vitamin-rich components, fresh chicken egg yolk (3%) as a source of lipids and sterols, phytoplankton (2%) as an additional source of micronutrients, and dH_2_O (70%) as the solvent. The diet was prepared as a syrup and applied by spraying onto the eggs of phytophagous species routinely maintained in our laboratory. This approach ensured that *A. swirskii* received both natural prey and supplemental artificial nutrition. The prepared syrup was stored at 5 °C for up to 14 days.

### 2.4. Experimental Design

To evaluate the vital parameters of *A. swirskii* reared on an artificial nutrient medium, experiments were conducted to assess fertility, viability, lifespan, and predation capacity under controlled laboratory conditions. At the initial stage, the primary objective was to obtain one-day-old eggs from females fed on ADs. For this purpose, 15 pairs of sexually mature *A. swirskii* adults were selected from the laboratory colony and transferred to specialized oviposition containers for egg collection. After 48 h, the collected eggs were transferred individually to rearing arenas for monitoring of nymph emergence.

The artificial diet was applied to prey eggs/cysts in the form of a syrup (5 µL per arena), ensuring a uniform coating and standardized feeding conditions across treatments. The number of eggs laid by each female over a 24 h period was recorded, along with the number of prey individuals consumed during the same time interval.

The experimental design included five diet treatments; each tested in triplicate:

C (Control): 1 ♀ + 1 ♂ *A. swirskii*+ AD + 5 (five) *C. lactis* eggs

D1: 1 ♀ + 1 ♂ *A. swirskii* + AD + decapsulated *A. salina* eggs (cysts)

D2: 1 ♀ + 1 ♂ *A. swirskii*+ AD + *S. cerealella* eggs

D3: 1 ♀ + 1 ♂ *A. swirskii*+ AD + *G. mellonella* eggs

D4: 1 ♀ + 1 ♂ *A. swirskii*+ AD + *T. turkestani* eggs + polyfloral pollen.

Each treatment consisted of five pairs of predatory mites (*n* = 5) per replicate, with three independent replicates, resulting in a total of 15 pairs per treatment. The sample size was chosen in line with previous studies on predatory mites [35,36], providing sufficient replication for statistical analysis (ANOVA) while ensuring practical feasibility.

Observations were conducted at 2 h intervals during the photoperiod throughout the experiment. The duration of the study was 14 days [37], which corresponds to the average developmental and reproductive cycle of *A. swirskii* at 25 °C (13–15 days) [40]. This period was therefore considered adequate to capture the key vital parameters of the species under laboratory conditions.

### 2.5. Vitality Indices Calculation

#### 2.5.1. Determination of Fertility

The fertility of *A. swirskii* females was assessed under laboratory conditions when fed on different artificial diets (ADs). To ensure comparability between treatments, egg production was recorded over a fixed period of two days. The mean daily oviposition per female was calculated using the following equation:

(1) $$Pl=\frac{N}{n\times t}$$

where Pl—mean number of eggs laid per female per day (pcs.); N—total number of eggs laid by all females during the observation period (pcs.); n—initial number of females placed in the rearing arena (pcs.); t—duration of the observation period (days, here t = 2). This value represents short-term daily oviposition and was used as a comparative index between diets. It does not reflect the complete lifetime fertility of females.

#### 2.5.2. Determination of Egg Hatching Rate

Egg hatching was determined as the proportion of eggs that successfully developed into viable nymphs. The hatching rate was calculated as:

(2) $$H=\frac{N_{h}}{N}\times 100\%$$

where H—egg hatching rate (%); N_h_—number of viable nymphs hatched from eggs laid over the two-day period (pcs.); N—total number of eggs laid during the same period (pcs.). This parameter strictly reflects egg-to-nymph emergence and was not conflated with survival to the adult stage.

#### 2.5.3. Determination of Adult Survival (Lifespan)

The longevity of females was monitored by recording natural mortality throughout the experiment. The average lifespan was calculated according to the formula:

(3) $$LS=\frac{N_{1}+N_{2}+\ldots +N_{n}}{F}$$

where LS—mean female lifespan (days); N_1_, N_2_ … N_n_—lifespan of each individual female (days); F—total number of females observed (pcs.). This index characterizes adult survival and longevity independently from fertility and hatching rate.

#### 2.5.4. Determination of Feeding Intensity in First-Generation *Amblyseius swirskii*

In this experiment, the feeding intensity of first-generation (*F*_1_) individuals of *A. swirskii*, obtained from parental cohorts maintained on artificial nutrient media of different compositions, was evaluated. As a standardized food substrate, *C. lactis* eggs were offered to the *F*_1_ nymphs. Each AD treatment was tested in three biological replicates.

Voracity was calculated using the following equation:

(4) $$Pr=\frac{\left(M-m\right)}{2n}$$

where Pr—number of spider mites consumed per female per day; M—initial number of spider mites placed in the arena (pcs.);m—number of remaining spider mites after 24 h (pcs.); n—number of predatory females in the arena (pcs.) [41].

Observations were conducted daily over a period of five consecutive days. During each observation period, the number of *C. lactis* eggs consumed by *A. swirskii* nymphs was recorded using a stereomicroscope. The data obtained were used to construct dynamic series illustrating changes in feeding intensity as a function of the parental diet administered during the previous generation.

### 2.6. Genetic Identification Based on ITS Region of rDNA

#### 2.6.1. DNA Extraction

Genomic DNA was extracted from individual mites using the DNeasy Blood and Tissue Kit (QIAGEN, Hilden, Germany) following the manufacturer’s protocol. The quality of the extracted DNA was assessed by electrophoresis on a 1% agarose gel.

#### 2.6.2. PCR Amplification

Polymerase chain reaction (PCR) was carried out using universal primers targeting the Internal Transcribed Spacer (ITS) region of ribosomal DNA (rDNA), covering ITS1, 5.8S, and ITS2. The primers used were ITS1 (forward): 5′-AGAGGAAGTAAAAGTCGTAACAAG-3′ and ITS2 (reverse): 5′-ATATGCTTAAATTCAGGGGG-3′, as described by Navajas et al. [42].

PCR was performed in a 20 μL reaction volume containing 4 μL of 5× HF reaction buffer (Thermo Scientific, Waltham, MA, USA), 1 μL of dNTPs, 0.5 μL of each primer, 0.2 μL of Hot Start Phusion High-Fidelity DNA Polymerase (Thermo Scientific, Waltham, MA, USA), and 2 μL of template DNA. The amplification was performed on a SimpliAmp Thermo Cycler (Life Technologies Corporation, Singapore) with the following thermal profile: initial denaturation at 98 °C for 30 s; 30 cycles of 98 °C for 10 s, 57 °C for 30 s, and 72 °C for 30 s; followed by a final extension at 72 °C for 10 min. PCR products were visualized on a 1% agarose gel to confirm successful amplification.

#### 2.6.3. ITS Gene Sequencing

Positive amplicons were purified using EXOSAP-IT™ PCR product purification reagent (Thermo Scientific, Waltham, MA, USA) and sequenced using the BigDye Terminator v3.1 Cycle Sequencing Kit. Sequencing was performed in both directions with the ITS1 and ITS2 primers using a 3500xL Genetic Analyzer (Applied Biosystems, Waltham, MA, USA), following the Sanger method.

The ITS gene sequences of *A. swirskii* were confirmed using BLASTN https://www.ncbi.nlm.nih.gov/ (accessed on 29 July 2025) [43] in the GenBank database of the National Center for Biotechnology Information (NCBI). Additional *A. swirskii* ITS sequences from various global regions were retrieved from GenBank, and multiple sequence alignments were performed using Clustal W v2.1. A phylogenetic tree was constructed using the Maximum Likelihood method in MEGA12 [44].The reliability of the tree clades was evaluated using bootstrap analysis with 1000 replicates, and all codon positions containing gaps or missing data were excluded from the analysis.

### 2.7. Statistical Analysis

All statistical analyses were conducted using R Studio (v.4.3.0). Prior to hypothesis testing, data were examined for normality using the Shapiro–Wilk test and for homogeneity of variances using Levene’s test [45].

For datasets meeting the assumptions of normality and homoscedasticity, a two-way analysis of variance (ANOVA) was performed, with diet (five levels: C, D1, D2, D3, D4) and time (days of observation) as fixed factors. Post hoc comparisons were carried out using Tukey’s HSD test. In cases where only two treatments were compared, Student’s t-test was applied.

When assumptions of normality or homogeneity were not satisfied, data were analyzed using the nonparametric Kruskal–Wallis test, followed by Mann–Whitney U tests for pairwise comparisons.

Categorical variables, including survival and sex ratio of progeny, were analyzed using Pearson’s Chi-square test. Statistical significance was determined at *p* < 0.05.

## 3. Results

### 3.1. Development and Fertility of A. swirskii Under Different Feeding Regimes

In this experiment, the effects of various feeding regimes on the developmental duration and overall biological activity of the predatory mite *A. swirskii* were evaluated under laboratory conditions. The study aimed to identify an optimal substitute for the natural diet (control: *C. lactis*) to support the effective mass rearing of this biological control agent. For each feeding treatment, the durations of the larval, nymphal, pre-imaginal, and imaginal stages were recorded (Table 1).

As shown in Table 1, the duration of the larval stage of *A. swirskii* remained consistent across all diet treatments, averaging approximately 1.0 day. No statistically significant differences were detected among the treatments at this stage (*p* > 0.05), indicating a negligible effect of diet composition on the progression of the larval phase.

A similar pattern was observed for the nymphal stage, with durations ranging from 1.9 to 2.5 days depending on the diet. Slightly prolonged development was noted in D1 (decapsulated *A. salina* cysts); however, the differences were not statistically significant (*p* > 0.05), suggesting limited influence of the protein composition of the diet on nymphal development.

In contrast, the total pre-imaginal development duration was significantly affected by diet type. The longest duration was recorded in D1 (decapsulated *A. salina* cysts) at 3.6 ± 0.2 days, which was significantly longer than that observed in D2 (*S. cerealella* eggs, 2.9 ± 0.1 days; *p* < 0.05). Treatments sharing the same letter in Table 1 did not differ significantly, while those with different letters (e.g., *a* vs. *b*) showed statistically significant differences according to Tukey’s HSD test. The D3 diet (*G. mellonella* eggs, 3.3 ± 0.1 days) yielded an intermediate value. The control (*C. lactis* eggs, 3.1 ± 0.1 days) and D4 (*Tetranychus turkestani* eggs + polyfloral pollen, 2.8 ± 0.1 days) treatments exhibited moderate durations, with no statistically significant differences from D2.

Diet composition also had a notable impact on adult longevity. The longest lifespans were recorded in D1 and D3 (10.5 ± 0.3 and 10.3 ± 0.3 days, respectively), both significantly exceeding the shortest lifespan observed in D2 (9.6 ± 0.2 days; *p* < 0.05). The control (9.9 ± 0.2 days) and D4 (10.4 ± 0.3 days) treatments yielded intermediate values, which did not differ significantly from the other treatments.

Overall, the most rapid development of *A. swirskii* was observed with D2 (*S. cerealella* eggs) and D4 (*T. turkestani* eggs), although these were associated with reduced (D2) or moderate (D4) adult longevity. Conversely, D1 (decapsulated *A. salina* cysts) resulted in slower pre-imaginal development but significantly enhanced adult longevity. A similar, though less pronounced, effect was observed with D3 (*G. mellonella* eggs).

These findings suggest that optimization of ADs for mass rearing of *A. swirskii* should consider both the rate of development and adult lifespan, as both parameters critically influence population productivity. Based on the combined performance metrics, the D1 diet was identified as the most promising option for maintaining high biological efficiency in artificial rearing systems.

**Figure 1:**
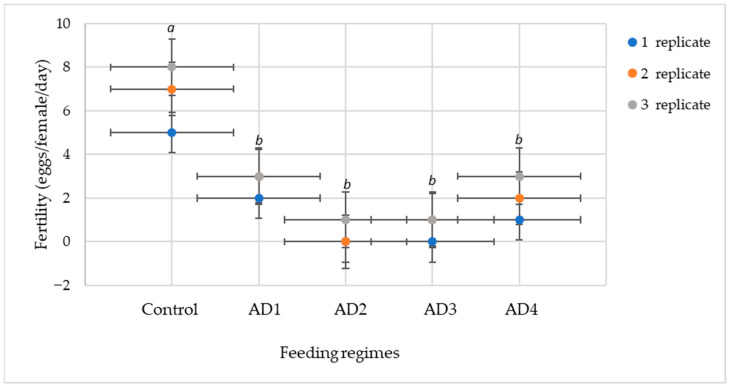
Fertility of *Amblyseius swirskii* females under different diets (Control—*C. lactis*; AD1—decapsulated *A. salina* cysts; AD2—*S. cerealella* eggs; AD3—*G. mellonella* eggs; AD4—*T. turkestani* eggs + polyfloral pollen). Different lowercase letters above the bars indicate statistically significant differences among treatments according to Tukey’s HSD test (*p* < 0.05). Error bars represent standard deviations.

In addition to developmental duration and adult longevity, the reproductive performance of *A. swirskii* females was evaluated under the various diet treatments. Figure 1 illustrates the mean daily fertility recorded for each feeding regime.

As shown in Figure 1, the control diet (*C. lactis* eggs) resulted in the highest fertility, averaging 6.6 eggs per female per day. Among the artificial diets, AD4 (2.0 eggs/day) and AD2 (0.3 eggs/day) supported moderate oviposition, whereas AD1 (2.6 eggs/day) and AD3 (0.7 eggs/day) showed the lowest fertility. Statistically significant differences (*p* < 0.05) between the control and all artificial diets are shown in Figure 1 by different lowercase letters.

Taken together, these results indicate that diet composition strongly affects the reproductive capacity of *A. swirskii*. While natural prey provided the highest fertility, AD4 demonstrated the most favorable balance between reproduction and development, whereas AD1 prolonged adult longevity but at the expense of reduced egg production. These findings suggest that artificial diet optimization for mass rearing must account for both developmental performance and fertility to maximize population growth potential.

### 3.2. Analysis of Nymph Emergence

This study further assessed the effect of various nutrient media formulations on the hatching success of eggs laid by the phytoseiid predatory mite *A. swirskii*.

#### 3.2.1. Nymph Hatching

**Figure 2:**
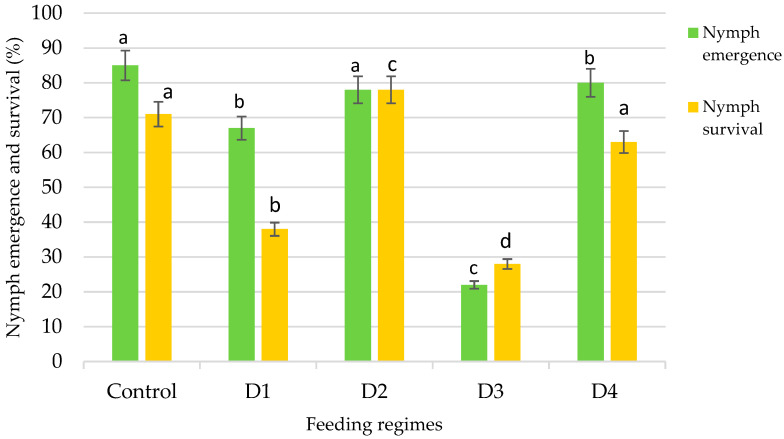
Emergence and survival of *Amblyseius swirskii* nymphs under different diet treatments (Control—*C. lactis*; AD1—decapsulated *A. salina* cysts; AD2—*S. cerealella* eggs; AD3—*G. mellonella* eggs; AD4—*T. turkestani* eggs + polyfloral pollen). Bars represent mean values ± SD. Different lowercase letters above the bars indicate statistically significant differences among treatments according to Tukey’s HSD test (*p* < 0.05).

Nymph emergence varied slightly depending on the type of diet (Figure 2). In the control treatment, where *C. lactis* eggs were used as feed, nymph hatching reached 85%. Under treatment D4, nymph hatching was 80%, while treatments D2 and D1 showed hatching rates of 78% and 67%, respectively.

When feeding on the D3 variant (*G. mellonella* eggs), nymph hatching was recorded at 22%, which was similar to the value observed for D2 (78%) and corresponded to the lowest overall survival rates under this treatment. These findings indicate the low suitability of this diet and suggest that it should be excluded from further studies. The most promising result in terms of nymph emergence was recorded for the D4 treatment (80%), which closely matched the efficacy of the standard laboratory diet.

#### 3.2.2. Nymph Survival

Survival of *A. swirskii* varied significantly depending on the diet provided (Figure 2). The lowest survival rate was observed with the diet containing *G. mellonella* eggs, indicating its low nutritional value or a potentially adverse effect on the developmental stages of the predatory mite.

The highest survival rate (78%) was recorded for treatment D2 (*S. cerealella* eggs), which was statistically significantly higher than the values obtained for all other treatments (*p* ≤ 0.05). Survival rates for the *T. turkestani* diet (D4) and the control diet (*C. lactis* eggs) were 63% and 71%, respectively, with no statistically significant difference between these two treatments.

The results indicate that *S. cerealella* eggs (D2) represent the most nutritionally favorable diet for *A. swirskii*. In contrast, the diet based on *A. salina* eggs (D1) proved to be the least suitable. These findings have important implications for optimizing the rearing conditions required for the efficient mass production of *A. swirskii* as a biological control agent in plant protection.

### 3.3. Feeding Dynamics and Lifespan of Amblyseius swirskii on Different Diets

Voracity is one of the fundamental criteria for evaluating the biological effectiveness of predatory mites. In this study, a detailed assessment was conducted on the voracity of first-generation (*F*_1_) individuals of *A. swirskii*, derived from parental cohorts reared on artificial nutrient media of varying compositions.

During the experiment, *F*_1_ nymphs of *A. swirskii* were provided with *C. lactis* eggs as a standardized food substrate (C treatment). The trial included three replicates, and food consumption was recorded daily over a period of five (5) days to monitor changes in voracity dynamics.

**Figure 3:**
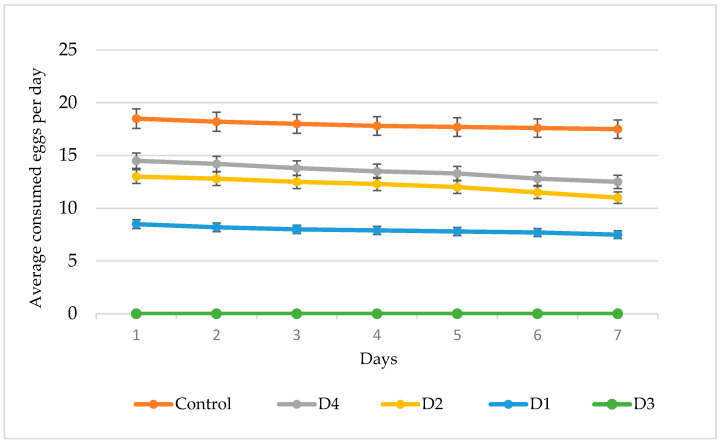
Voracity of F_1_ generation of *Amblyseius swirskii* females after different parental diets (Control—*C. lactis*; AD1—decapsulated *A. salina* cysts; AD2—*S. cerealella* eggs; AD3—*G. mellonella* eggs; AD4—*T. turkestani* eggs + polyfloral pollen; mean ± SD, *n* = 15).

The results revealed a clear dependence of the feeding intensity of predatory mites on the composition of the artificial diets used during the parental rearing phase. Under optimal climatic conditions and access to the standard prey (*C. lactis* eggs), the average daily egg consumption of *A. swirskii* nymphs in the F_1_ generation ranged between 7.5 and 18.5 eggs per day (Figure 3).

As illustrated in Figure 3, the transfer of *A. swirskii* progeny to artificial diets resulted in a marked reduction in feeding intensity compared to the control group fed on *C. lactis* eggs. In the control treatment, feeding activity remained consistently high throughout the observation period, ranging from 17.5 to 18.5 eggs per day, with an overall average of 18.0 eggs per day.

The use of alternative diets in the parental generation led to a progressive decline in trophic activity in the subsequent generation. In D4 (*T. turkestani* eggs), the average daily prey consumption decreased from 14.5 and 14.2 eggs on days 1 and 2, respectively, to 12.5 eggs by day 7,with an overall average of 13.5 eggs per day across the entire experimental period. A more pronounced decline was observed in the D2 treatment (*S. cerealella* eggs), where consumption dropped from 13.0 to 11.0 eggs/day (mean: 12.0 eggs/day). The lowest values were recorded in D1 (decapsulated *A. salina* cysts), beginning at 8.5 eggs/day and stabilizing between 7.5 and 8.0 eggs/day during the subsequent days.

It should be noted that in the case of diet D3 (*G. mellonella* eggs), both the emergence and survival of nymphs were markedly reduced compared to other treatments. As a result, the number of viable individuals was insufficient for a reliable assessment of their feeding activity. This finding indicates the low suitability of this diet for the rearing of *A. swirskii* and highlights the need to exclude it from further studies on the optimization of artificial diets.

The dynamics of prey consumption demonstrated that the highest feeding intensity was maintained on the control diet (*C. lactis*) and partially preserved on diet D4. In contrast, D1 resulted in the lowest feeding levels.

### 3.4. Molecular Genetic Identification of A. swirskii

Genomic DNA isolated from *A. swirskii* was successfully amplified using species-specific primers targeting the ITS region of ribosomal DNA. The resulting amplicons were sequenced and aligned against sequences in the NCBI GenBank database for identification.

**Figure 4:**
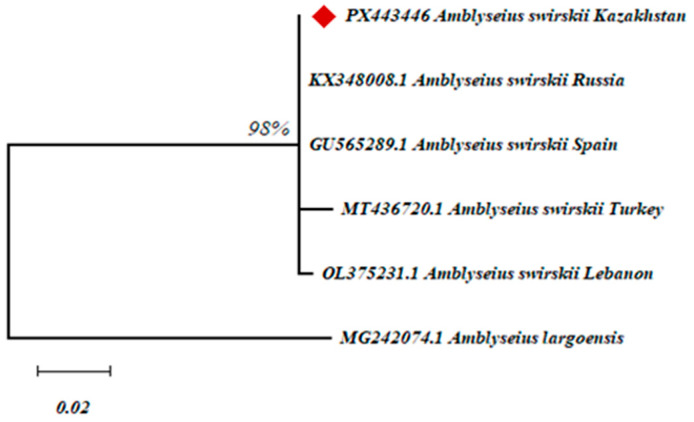
Phylogenetic tree constructed based on ITS rDNA sequences, illustrating the relationships between the studied *A. swirskii* isolate and reference taxa from the GenBank database.

The phylogenetic tree (Figure 4) constructed based on ITS sequences revealed that the Kazakh isolate of *A. swirskii* (marked with a red diamond) clustered within a strongly supported clade (bootstrap = 98%) alongside reference isolates from Spain, Turkey, Russia, and Lebanon, confirming its taxonomic affiliation with the species *A. swirskii*. The high degree of genetic similarity among geographically distinct populations indicates strong genetic conservatism at the ITS region level. The phylogenetic distance between *A. swirskii* and *A. largoensis*, observed as a distinctly separate branch, further supports the taxonomic isolation of the studied group. The resulting sequence was deposited in GenBank under the accession number PX443446.

## 4. Discussion

The results of this study confirm that the lifespan and performance of *Amblyseius swirskii* are closely dependent on the nutritional adequacy and composition of the diet. A balanced diet plays a pivotal role in supporting the physiological functions and reproductive capacity of this species. Among all tested diets, natural feeding on the food mite *C. lactis* yielded the highest survival rates and trophic activity. This is likely due to the comprehensive profile of essential nutrients, proteins, lipids, vitamins, and minerals present in *C. lactis* in proportions optimally suited to the nutritional demands of predatory mites.

In this study, variation in the longevity and survival of *A. swirskii* females was observed across diet treatments. Diet D2 (AD + *S. cerealella* eggs) demonstrated the most favorable outcomes among artificial options, with significantly higher survival rates compared to other tested diets. Diet D1 (AD + *A. salina* cysts) tended to prolong adult lifespan, although differences were not statistically significant when compared to some other treatments. Diet D3 (AD + *G. mellonella* eggs) showed consistently poor performance, with significantly reduced survival and fertility. These results indicate that while certain artificial diets may support development and survival, they generally do not fully match the performance achieved with natural feeding on *C. lactis*.

Feeding intensity in the F_1_ generation further highlighted the impact of parental diet. Nymphs originating from parents reared on artificial diets exhibited reduced voracity compared to the control, with D1 and D2 showing notably lower prey consumption. This reduction was statistically significant in comparison with the control diet, whereas the differences among artificial diet treatments largely represented tendencies rather than strong statistical contrasts. These observations are consistent with previous findings, where parental nutrition influenced the feeding behavior and fitness of offspring [46].

In addition to feeding activity, the longevity of F_1_ nymphs was also affected by diet composition during subsequent observations. In the D1 group, females lived 12–15 days, indicating that this formulation met basic physiological requirements. The D2 diet extended longevity to 15–18 days. The shortest lifespan (10–13 days) was observed in the D3 treatment, attributed to inadequate nutrient content. The D4 diet supported a lifespan of 12–16 days. The control group, fed *C. lactis* eggs, exhibited the longest adult lifespan, ranging from 18 to 22 days, confirming the optimality of natural prey for sustaining the biological potential of *A. swirskii*.

Our findings align with earlier studies. For instance, Abou-Awad et al. [47] reported that *A. swirskii* and *A. gossipi* could complete their life cycles on artificial diets containing yeast, milk, amino acids, and sucrose, though egg-laying was reduced compared to natural prey. Ogawa and Osakabe [37] showed that supplementing artificial diets for *Neoseiulus californicus* with egg yolk and yeast extract improved longevity, though development was still slower than with live prey. In our case, diet D2 supported faster pre-imaginal development and relatively high survival, while diet D1 showed a tendency toward longer adult lifespan.

Overall, these results reaffirm the conclusion that *A. swirskii* can complete its life cycle on artificial diets; however, significant improvements are required to achieve performance levels comparable to natural prey. A particularly promising direction involves the integration of high-protein components, such as insect eggs and hemolymph, with vitamin and mineral supplements, as well as probiotics. This multifactorial approach may enhance both survival and reproductive potential of the predatory mite, thereby contributing to the development of efficient and scalable systems for its mass rearing and application in biological control programs.

In this study, we applied classical methods (ANOVA, Tukey’s HSD, *t*-tests, and nonparametric tests), which were suitable for our experimental design and replication level. However, these approaches do not account for potential random effects or non-independence among replicates. More advanced approaches, such as Generalized Linear Mixed Models (GLMM), could provide greater flexibility for hierarchical data and non-normal distributions. Future studies applying GLMM are recommended to strengthen inference, and our conclusions should be interpreted within the scope of the statistical tools used.

## 5. Conclusions

This study presents the first comprehensive evaluation in Kazakhstan of the effects of artificial diets on the main biological parameters of the predatory mite *Amblyseius swirskii*. The results confirmed that diet composition plays a decisive role in development rate and reproductive potential. The highest survival rate (78%) and accelerated development were obtained with *S. cerealella* eggs, while decapsulated *A. salina* cysts prolonged adult longevity. The diet combining *T. turkestani* eggs with pollen provided balanced performance, whereas *G. mellonella* eggs proved least suitable for mass rearing.

Overall, *A. swirskii* can complete its life cycle on artificial diets, but further refinement of formulations is needed for efficient mass production. The findings can be applied in the development of technological protocols for the mass rearing of predatory mites and their use in the biological protection of vegetable crops, thereby reducing the application of insecto-acaricides in protected cultivation.

## Figures and Tables

**Table 1:** Duration of developmental stages of *A. swirskii* under different feeding regimes (days; mean ± SD, *n* =15).

Development Stage	C	D1	D2	D3	D4
Larval	1.0 ± 0.1	1.1 ± 0.1	0.9 ± 0.1	1.0 ± 0.1	0.9 ± 0.1
Nymphal	2.1 ± 0.1	2.5 ± 0.2	2.0 ± 0.1	2.3 ± 0.1	1.9 ± 0.1
Pre-imaginal	3.1 ± 0.1 ^ab^	3.6 ± 0.2 ^a^	2.9 ± 0.1 ^b^	3.3 ± 0.1 ^a^	2.8 ± 0.1 ^b^
Imaginal	9.9 ± 0.2 ^ab^	10.5 ± 0.3 ^a^	9.6 ± 0.2 ^b^	10.3 ± 0.3 ^a^	10.4 ± 0.3 ^a^
HSD	−	−	0.3	−	0.4

Different lowercase letters indicate statistically significant differences between means according to Tukey’s HSD test (*p* < 0.05).

## Data Availability

The original contributions presented in this study are included in the article. Further inquiries can be directed to the corresponding author.
